# Performance improvement via bagging in probabilistic prediction of chaotic time series using similarity of attractors and LOOCV predictable horizon

**DOI:** 10.1007/s00521-017-3149-7

**Published:** 2017-07-15

**Authors:** Shuichi Kurogi, Mitsuki Toidani, Ryosuke Shigematsu, Kazuya Matsuo

**Affiliations:** Department of Control Engineering, Kyushu Institute of Technology, Tobata, Kitakyushu, Fukuoka, 804-8550 Japan

**Keywords:** Probabilistic prediction of chaotic time series, Long-term unpredictability, Attractors of chaotic time series, Leave-one-out cross-validation, Estimation of predictable horizon

## Abstract

Recently, we have presented a method of probabilistic prediction of chaotic time series. The method employs learning machines involving strong learners capable of making predictions with desirably long predictable horizons, where, however, usual ensemble mean for making representative prediction is not effective when there are predictions with shorter predictable horizons. Thus, the method selects a representative prediction from the predictions generated by a number of learning machines involving strong learners as follows: first, it obtains plausible predictions holding large similarity of attractors with the training time series and then selects the representative prediction with the largest predictable horizon estimated via LOOCV (leave-one-out cross-validation). The method is also capable of providing average and/or safe estimation of predictable horizon of the representative prediction. We have used CAN2s (competitive associative nets) for learning piecewise linear approximation of nonlinear function as strong learners in our previous study, and this paper employs bagging (bootstrap aggregating) to improve the performance, which enables us to analyze the validity and the effectiveness of the method.

## Introduction

So far, a number of methods for time series prediction have been studied (cf. [1, 2]), and our methods have awarded 3rd and 2nd places in the competitions of time series prediction held at IJCNN’04 [3] and ESTSP’07 [4], respectively. Our methods have used model selection methods evaluating MSE (mean square prediction error) for holdout and/or cross-validation datasets. Recently, we have developed several model selection methods for chaotic time series prediction [5, 6]. The method in [5] utilizes moments of predictive deviation as ensemble diversity measures for model selection in time series prediction and achieves better performance from the point of view of MSE than the conventional holdout method. The method in [6] uses direct multistep ahead (DMS) prediction to apply the out-of-bag (OOB) estimate of MSE. Although both methods have selected the models to generate good predictions on average, they cannot always have provided good predictions, especially when the horizon to be predicted is large. This is owing mainly to the fact that the MSE of a set of predictions is largely affected by a small number of predictions with short predictable horizons even if most of the predictions have long predictable horizons. This is because the prediction error of chaotic time series increases exponentially with the increase in time after the predictable horizon (see [6] for the analysis and [1] for properties of chaotic time series).

Instead of using model selection methods employing the estimation of the MSE, we have developed a method of probabilistic prediction of chaotic time series [7]. Here, from [8], the probabilistic prediction has come to dominate the science of weather and climate forecasting, mainly because the theory of chaos at the heart of meteorology shows that for a simple set of nonlinear equations (or Lorenz’s equations shown below) with initial conditions changed by minute perturbations, there is no longer a single deterministic solution and hence all forecasts must be treated as probabilistic. Although most of the methods shown in [8] use ensemble mean for representative forecast, our method in [7] (see below for details) uses an individual prediction selected from a set of plausible predictions for the representative because our method employs learning machines involving strong learners capable of making predictions with small error for a desirably long duration and we can see that ensemble mean does not work when the set of predictions for the ensemble involves a prediction with short predictable horizon. This is owing mainly to the exponential increase in prediction error of chaotic time series after the predictable horizon (see Sect. 3.2 for details)

Thus, instead of using ensemble mean, our method in [7] firstly selects plausible predictions by means of evaluating the similarity of attractors between training and predicted time series and then obtains the representative prediction by means of LOOCV (leave-one-out cross-validation) to select the prediction with longer predictable horizon. Comparing with our previous methods using the MSE for model selection [5, 6], the method in [7] has an advantage that it is capable of selecting the representative prediction from plausible predictions for each start time of prediction and providing the estimation of predictable horizon. Furthermore, it has achieved long predictable horizons on average. However, there are several cases where the method selects representative prediction with short predictable horizon, although there are plausible predictions with longer predictable horizons.

To overcome this problem, this paper tries to improve the performance of learning machines by using bagging (bootstrap aggregating) method and show the analysis of LOOCV predictable horizon. Here, the bagging is known to use ensemble mean to have an ability to reduce the variance of predictions by single learning machines, and then, we can expect that the performance in time series prediction becomes more stable and higher. Note that, in this paper, the bagging ensemble is employed for iterated one-step-ahead (IOS) prediction of time series, and we deal with probabilistic prediction as an ensemble of longer-term predictions. Furthermore, we use CAN2 (competitive associative net 2) as a learning machine (see [3] for the details of CAN2), where CAN2 has been introduced for learning piecewise linear approximation of nonlinear function and the performance has been shown in evaluating predictive uncertainty challenge [9], where our method has been awarded the first place in regression problems. The CAN2 has been used in our methods [3, 4] for the competitions of time series predictions shown above.

We show the present method of probabilistic prediction of chaotic time series in Sect. 2, experimental results and analysis in Sect. 3, and the conclusion in Sect. 4.

## Probabilistic prediction of chaotic time series

### IOS prediction of chaotic time series

Let $y_t \,(\in {\mathbb {R}})$ denote a chaotic time series for a discrete time $t=0,1,2,\ldots$ satisfying

1 $$\begin{aligned} y_t=r(\varvec{x}_t)+ e(\varvec{x}_t), \end{aligned}$$

where $r(\varvec{x}_t)$ is a nonlinear target function of a vector $\varvec{x}_t=(y_{t-1},y_{t-2},\ldots ,y_{t-k})^{\mathrm{T}}$ generated by *k*-dimensional delay embedding from a chaotic differential dynamical system (see [1] for the theory of chaotic time series). Here, $y_t$ is obtained not analytically but numerically, and then, $y_t$ involves an error $e(\varvec{x}_t)$ owing to an executable finite calculation precision. This indicates that there are a number of plausible target functions $r(\varvec{x}_t)$ with allowable error $e(\varvec{x}_t)$. Furthermore, in general, a time series generated with higher precision has small prediction error for longer duration of time from the initial time of prediction. Thus, let a time series generated with a high precision (or 128-bit precision; see Sect. 3 for details), be ground truth time series ${y}^{[{\mathrm{\tiny gt}}]}_{t}$, while we examine predictions generated with standard 64-bit precision.

Let $y_{t:h}=y_t \,y_{t+1}\ldots y_{t+h-1}$ denote a time series with the initial time *t* and the horizon *h*. For a given training time series $y_{t_{\mathrm{\tiny g}}:h_{\mathrm{\tiny g}}}(={y}^{[{\mathrm{\tiny train}}]}_{t_{\mathrm{\tiny g}}:h_{\mathrm{\tiny g}}})$, we are supposed to predict succeeding time series $y_{t_{\mathrm{\tiny p}}:h_{\mathrm{\tiny p}}}$ for $t_{\mathrm{\tiny p}}\ge t_{\mathrm{\tiny g}}+h_{\mathrm{\tiny g}}$. Then, we make the training dataset ${D}^{[{\mathrm{\tiny train}}]}_{}=\{(\varvec{x}_{t},y_{t})\mid t \in {I}^{[{\mathrm{\tiny train}}]}_{}\}$ for ${I}^{[{\mathrm{\tiny train}}]}_{}=\{t\mid t_{\mathrm{\tiny g}}+k\le t< t_{\mathrm{\tiny g}}+h_{\mathrm{\tiny g}}\}$ to train a learning machine. After the learning, the machine executes IOS prediction by

2 $$\begin{aligned} \hat{y}_{t}=f({\varvec{x}}_{t}) \end{aligned}$$

for $t=t_{\mathrm{\tiny p}},t_{\mathrm{\tiny p}+1},\ldots$, recursively, where $f({\varvec{x}}_t)$ denotes prediction function of ${\varvec{x}}_t=({x}_{t1},{x}_{t2},\ldots ,{x}_{tk})$ whose elements are given by ${x}_{tj}={y}_{t-j}$ for $t-j< t_{\mathrm{\tiny p}}$ and ${x}_{tj}=\hat{y}_{t-j}$ for $t-j\ge t_{\mathrm{\tiny p}}$. Here, we suppose that $y_{t}$ for $t<t_{\mathrm{\tiny p}}$ is known as the initial state for making the prediction ${\hat{y}}_{t_p:h_p}$. As explained above, we execute the prediction with standard 64-bit precision, and we may say that there are a number of plausible prediction functions $f(\varvec{x}_t)$ with small error for a duration of time from the initial time of prediction by means of using strong learning machines.

### Single CAN2 and the bagging for IOS prediction

We use CAN2 as a learning machine. A single CAN2 has *N* units. The $j$th unit has a weight vector $\varvec{w}_{j}\triangleq (w_{{j}1},\ldots ,w_{{j}k})^{\mathrm{T}}\in {\mathbb {R}}^{k\times 1}$ and an associative matrix (or a row vector) $\varvec{M}_{j}\,\triangleq \,(M_{{j}0},M_{{j}1},\ldots ,M_{{j}k})\in {\mathbb {R}}^{1\times (k+1)}$ for ${j}\in I^{N} \triangleq \{1,2,\ldots ,N\}$. The CAN2 after learning the training dataset ${D}^{[\mathrm{\tiny train}]}_{}=\{(\varvec{x}_t,y_t)\mid t\in {I}^{[{\mathrm{\tiny train}}]}_{}\}$ approximates the target function $r(\varvec{x}_t)$ by

3 $$\begin{aligned} \widehat{y}_t =\widetilde{y}_{c(t)} =\varvec{M}_{c(t)}\widetilde{\varvec{x}}_t, \end{aligned}$$

where $\widetilde{\varvec{x}}_t\, \triangleq \,(1,\varvec{x}_t^{\mathrm{T}})^{\mathrm{T}}\in {\mathbb {R}}^{(k+1)\times 1}$ denotes the (extended) input vector to the CAN2, and $\widetilde{y}_{c(t)}=\varvec{M}_{c(t)}\widetilde{\varvec{x}}_t$ is the output value of the *c*(*t*)th unit of the CAN2. The index *c*(*t*) indicates the unit who has the weight vector $\varvec{w}_{c(t)}$ closest to the input vector $\varvec{x}_t$, or $c(t)\triangleq \mathop{{\mathrm{argmin}}}\nolimits_{{j}\in I^N} \Vert \varvec{x}_t-\varvec{w}_{j}\Vert.$ Note that the above prediction performs piecewise linear approximation of $y=r(\varvec{x})$ and *N* indicates the number of piecewise linear regions. We use the learning algorithm shown in [10] whose high performance in regression problems has been shown in evaluating predictive uncertainty challenge [9].

We obtain bagging prediction by means of using a number of single CAN2s as follows (see [11, 12] for details); let ${D}^{[n\alpha ^{\sharp },{j}]}_{}=\{(\varvec{x}_{t},y_{t})\mid \, t\in {I}^{[n\alpha ^{\sharp },{j}]}_{})\}$ be the ${j}$th bag (multiset, or bootstrap sample set) involving $n\alpha$ elements, where the elements in ${D}^{[n\alpha ^{\sharp },{j}]}_{}$ are resampled randomly with replacement from the training dataset ${D}^{[{\mathrm{\tiny train}}]}_{}$ involving $n=|{D}^{[{\mathrm{\tiny train}}]}_{}|$ elements. Here, $\alpha \,({>}0)$ indicates the bag size ratio to the given dataset, and ${j}\in {J}^{[{\mathrm{\tiny bag}}]}_{}\triangleq \{1,2,\ldots ,b\}$. Here, note that $\alpha =1$ is used in many applications (see [12, 13]), which we use in the experiments shown below after the tuning of $\alpha$ (see [12] for validity and effectiveness of using variable $\alpha$). Using multiple CAN2s employing *N* units after leaning ${D}^{[n\alpha ^{\sharp }]}_{,}{j}$, which we denote ${\theta }^{[j]}_{N} \,(\in {\varTheta }_{N}\triangleq \{{\theta }^{[j]}_{N}\mid {j}\in {J}^{[{\mathrm{\tiny bag}}]}_{}\})$, the bagging for predicting the target value $r_{t_c}=r(\varvec{x}_{t_c})$ is done by

4 $$\begin{aligned} {\hat{y}}^{[{\theta }^{[{\mathrm{bag}}]}_{N}]}_{t}\triangleq \frac{1}{b}\sum _{j \in {J}^{[{\mathrm{bag}}]}_{}} {\hat{y}}^{[j]}_{t} \equiv \left\langle {\hat{y}}^{[j]}_{t}\right\rangle _{j\in {J}^{[{\mathrm{bag}}]}_{}} \end{aligned}$$

where ${\hat{y}}^{[j]}_{t_c}\triangleq {\hat{y}}^{[j]}_{}(\varvec{x}_{t_c})$ denotes the prediction by the *j*th machine ${\theta }^{[j]}_{N}$. The angle brackets $\left\langle \cdot \right\rangle$ indicate the mean, and the subscript ${j}\in {J}^{[{\mathrm{bag}}]}_{}$ indicates the range of the mean. For simple expression, we sometimes use ${\langle \cdot \rangle }_{j}$ instead of ${\langle \cdot \rangle }_{{j}\in {J}^{[{\mathrm{bag}}]}_{}}$ in the following.

### Probabilistic prediction and estimation of predictable horizon

#### Similarity of attractors to select plausible predictions

First, we make a number of IOS predictions $\hat{y}_{t_p:h_p}={y}^{[{\theta }_{N}]}_{t_p:h_p}$ by means of learning machines or CAN2s, ${\theta }_{N}\in \varTheta _{}$, with different number *N* of units, where $\varTheta _{}$ indicates the set of all learning machines. We employ single and bagging CAN2s, which we denote ${\theta }^{[{\mathrm {single}}]}_{N}$ and ${\theta }^{[{\mathrm{bag}}]}_{N}$, respectively, if necessary. We suppose that there are a number of plausible prediction functions $f(\cdot )={f}^{[{\theta }_{N}]}_{}(\cdot )$, and we have to remove implausible ones. To have this done, we select the following set of plausible predictions:

5 $$\begin{aligned} {Y}^{[S_{\mathrm{\tiny th}}]}_{t_p:h_p}= \left\{ {y}^{[{\theta }_{N}]}_{t_p,h_p}\biggm | S\left( {y}^{[{\theta }_{N}]}_{t_p,h_p},{y}^{[{\mathrm{\tiny train}}]}_{t_g:h_g}\right) \ge S_{\mathrm{\tiny th}}, {\theta }_{N}\in \varTheta \right\} \end{aligned}$$

where

6 $$\begin{aligned} S\left( {y}^{[{\theta }_{N}]}_{t_p,h_p},{y}^{[{\mathrm{\tiny train}}]}_{t_g:h_g}\right) \triangleq \frac{\sum _i \sum _j {a}^{[{\theta }_{N}]}_{ij} {a}^{[{\mathrm{\tiny train}}]}_{ij} }{\sqrt{\sum _i \sum _j \left( {a}^{[{\theta }_{N}]}_{ij}\right) ^2} \sqrt{\sum _i \sum _j \left( {a}^{[{\mathrm{\tiny train}}]}_{ij}\right) ^2} } \end{aligned}$$

denotes the similarity of two-dimensional attractor (trajectory) distributions ${a}^{[{\theta }_{N}]}_{ij}$ and ${a}^{[{\mathrm{\tiny train}}]}_{ij}$ of time series ${y}^{[{\theta }_{N}]}_{t_p,h_p}$ and ${y}^{[{\mathrm{\tiny train}}]}_{t_g:h_g}$, respectively, and $S_{\mathrm{\tiny th}}$ is a threshold. Here, the two-dimensional attractor distribution, $a_{ij}$, of a time series $y_{t:h}$ is given by

7 $$\begin{aligned} a_{ij}=\sum _{s=t}^{t+h-1} \varvec{1}\left\{ \left\lfloor \frac{y_{s}-v_{0}}{\Delta _a}\right\rfloor =i \wedge \left\lfloor \frac{y_{s+1}-v_{0}}{\Delta _a}\right\rfloor =j \right\} , \end{aligned}$$

where $v_{0}$ is a constant less than the minimum value of $y_t$ for all time series and $\Delta _a$ indicates a resolution of the distribution. Furthermore, $\varvec{1}\{z\}$ is an indicator function equal to 1 if *z* is true, and 0 if *z* is false, and $\lfloor \cdot \rfloor$ indicates the floor function.

#### LOOCV measure to estimate predictable horizons

Let us define predictable horizon between two predictions ${y}^{[{\theta }_{N}]}_{t_p:h_p}$ and ${y}^{[{\theta }_{N'}]}_{t_p:h_p}$ in ${Y}^{[S_{\mathrm{\tiny th}}]}_{t_p:h_p}$ as

8 $$\begin{aligned} h\left( {y}^{[{\theta }_{N}]}_{t_p:h_p},{y}^{[{\theta }_{N'}]}_{t_p:h_p}\right) =\max \left\{ h\bigm |{\forall } s< h \le h_p; |{y}^{[{\theta }_{N}]}_{t_p+s} -{y}^{[{\theta }_{N'}]}_{t_p+s}|\le e_y \right\} , \end{aligned}$$

where $e_y$ indicates the threshold of prediction error to determine the horizon. Then, we employ LOOCV method to estimate predictable horizon of ${y}^{[{\theta }_{N}]}_{t_p:h_p}$ in ${Y}^{[S_{\mathrm{\tiny th}}]}_{t_p:h_p}$. Namely, we use

9 $$\begin{aligned} {\tilde{h}}^{[{\theta }_{N}]}_{t_p:h_p}&=h\left( {y}^{[{\theta }_{N}]}_{t_p:h_p},{Y}^{[S_{\mathrm{\tiny th}}]}_{t_p:h_p}\bigm \backslash \{{y}^{[{\theta }_{N}]}_{t_p:h_p}\}\right) \nonumber \\&=\left\langle h\left( {y}^{[{\theta }_{N}]}_{t_p:h_p},{y}^{[{\theta }_{N'}]}_{t_p:h_p}\right) \right\rangle _{{y}^{[{\theta }_{N'}]}_{t_p:h_p}\in {Y}^{[S_{\mathrm{\tiny th}}]}_{t_p:h_p}\bigm \backslash \{{y}^{[{\theta }_{N}]}_{t_p:h_p} \}}, \end{aligned}$$

which we call LOOCV measure of predictable horizon or LOOCV predictable horizon. Here, we expect that ${h}\left( {y}^{[{\theta }_{N}]}_{t_p:h_p},{Y}^{[S_{\mathrm{\tiny th}}]}_{t_p:h_p}\bigm \backslash \{{y}^{[{\theta }_{N}]}_{t_p:h_p}\right)$ and $h\left( {y}^{[{\theta }_{N}]}_{t_p:h_p},{y}^{[{\mathrm{\tiny gt}}]}_{t}\right)$ have positive correlation by means of assuming that ${Y}^{[S_{\mathrm{\tiny th}}]}_{t_p:h_p}$ involves a number of predictions neighboring ${y}^{[{\mathrm{\tiny gt}}]}_{t}$.

#### Probabilistic prediction involving longer LOOCV predictable horizons

Let a subset of plausible predictions involving longer LOOCV predictable horizons be

10 $$\begin{aligned} {Y}^{[H_{\mathrm{\tiny th}},S_{\mathrm{\tiny th}}]}_{t_p:h_p}= \left\{ {y}^{[\theta _{\sigma (i)}]}_{t_p:h_p}\biggm | \frac{i}{|{Y}^{[S_{\mathrm{\tiny th}}]}_{t_p:h_p}|} \le H_{\mathrm{\tiny th}} \right\} , \end{aligned}$$

where $\sigma (i)$ denotes the order of LOOCV predictable horizons satisfying ${\tilde{h}}^{[\theta _{\sigma (i)}]}_{t_p:h_p}\ge {\tilde{h}}^{[\theta _{\sigma (i+1)}]}_{t_p:h_p}$ for $i=1,2,\ldots ,|{Y}^{[S_{\mathrm{\tiny th}}]}_{t_p:h_p}|$. The threshold $H_{\mathrm{\tiny th}}\,(0< H_{\mathrm{\tiny th}} \le 1)$ indicates the ratio of the number of elements in ${Y}^{[H_{\mathrm{\tiny th}},S_{\mathrm{\tiny th}}]}_{t_p:h_p}$ and ${Y}^{[S_{\mathrm{\tiny th}}]}_{t_p:h_p}$, or $\left| {Y}^{[H_{\mathrm{\tiny th}},S_{\mathrm{\tiny th}}]}_{t_p:h_p}\right| =H_{\mathrm{\tiny th}}\left| {Y}^{[S_{\mathrm{\tiny th}}]}_{t_p:h_p}\right|$. Now, we derive the probability of the prediction ${y}_t$ for $t_p\le t< t_p+h_p$ as

11 $$\begin{aligned} p\left( v_i\le y_t<v_{i+1}\right) = \left\langle \varvec{1}\left\{ \left\lfloor \frac{{y}^{[\theta ]}_{t}-v_0}{\Delta _v}\right\rfloor =i\right\} \right\rangle _{\theta \in {\varTheta }^{[H_{\mathrm{\tiny th},S_{\mathrm{\tiny th}}}]}_{}} \end{aligned}$$

where ${\varTheta }^{[H_{\mathrm{\tiny th},S_{\mathrm{\tiny th}}}]}_{}$ is the set of parameters $\theta$ of learning machines which have generated ${y}^{[\theta ]}_{t_p:h_p}\in {Y}^{[H_{\mathrm{\tiny th}},S_{\mathrm{\tiny th}}]}_{t_p:h_p}$, and $\Delta _v$ denotes the resolution of $y_t$, and $v_i=i\Delta _v+v_0$ for $i=0,1,2,\ldots$. Note that the probability $p(v_i\le y_t\le v_{i+1})$ indicates how much the plausible predictions in ${Y}^{[H_{\mathrm{\tiny th}},S_{\mathrm{\tiny th}}]}_{t_p:h_p}$ take the values in between $v_i$ and $v_{i+1}$.

#### Representative prediction and estimation of predictable horizon

Now, we provide ${y}^{[\theta _{\sigma (1)}]}_{t_p:h_p}$ as a representative prediction, and an estimation of the predictable horizon ${h}^{[\theta _{\sigma (1)}]}_{t_p:h_p}=h\left( {y}^{[\theta _{\sigma (1)}]}_{t_p:h_p},{y}^{[{\mathrm{\tiny gt}}]}_{t_p:h_p}\right)$ as

12 $$\begin{aligned} {\hat{h}}^{[\theta _{\sigma (1)}]}_{t_p:h_p} =\min \left\{ h(y^{[\theta _{\sigma (1)}]}_{t_p:h_p},y^{[\theta ]}_{t_p:h_p})\bigm |\forall y^{\theta }_{t_p:h_p}\in Y^{[H_{th},S_{th}]}_{t_p:h_p} \bigm \backslash y^{[\theta _{\sigma (1)}]}_{t_p:h_p} \right\} , \end{aligned}$$

where we have to tune $H_{\mathrm{\tiny th}}$ from the point of view of accuracy and safeness. Here, the safe estimation of ${\hat{h}}^{[\theta _{\sigma (1)}]}_{t_p:h_p}$ indicates that ${\hat{h}}^{[\theta _{\sigma (1)}]}_{t_p:h_p}$ is smaller than or equal to the actual predictable horizon ${h}^{[\theta _{\sigma (1)}]}_{t_p:h_p}$, and we can see that ${\hat{h}}^{[\theta _{\sigma (1)}]}_{t_p:h_p}$ become safer with the increase in $H_{\mathrm{\tiny th}}$.

## Numerical experiments and analysis

### Experimental settings

**Fig. 1:**
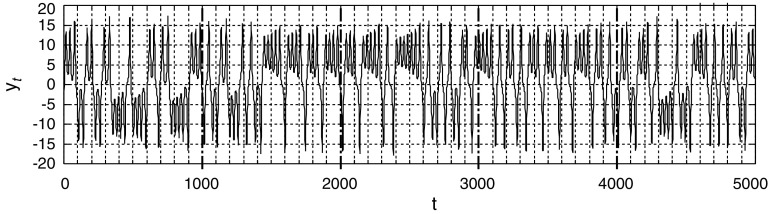
Lorenz time series $y_t$ for $t=0,1,2,\ldots ,4999$, or ground truth time series ${y}^{[{\mathrm{\tiny gt}}]}_{0:5000}$

We use the Lorenz time series, as shown in Fig. 1 and [6], obtained from the original differential dynamical system given by

13 $$\begin{aligned} \frac{\mathrm{d}x_c}{\mathrm{d}t_c}=\sigma (y_c-x_c), \quad \frac{\mathrm{d}y_c}{\mathrm{d}t_c}=-x_cz_c+rx_c-y_c, \quad \frac{\mathrm{d}z_c}{\mathrm{d}t_c}=x_cy_c-bz_c, \end{aligned}$$

for $\sigma =10$, $r=28$ and $b=8/3$. Here, we use $t_c$ for continuous time and $t \,(=0,1,2,\ldots )$ for discrete time related by $t_c=tT$ with the sampling time or the embedding delay $T=25$ ms. We have generated the time series ${y}^{[{\mathrm{\tiny gt}}]}_{t}=x_c(t T)$ for $t=1,2,\ldots ,5000$ from the initial state $(x_c(0),y_c(0),z_c(0))=(-8,8,27)$ via the fourth-order Runge–Kutta method with step size $\Delta t=10^{-4}$ and $r=$ 128-bit precision of GMP (GNU multiprecision library).

Using ${y}^{[{\mathrm{\tiny train}}]}_{t_g:h_g}={y}^{[{\mathrm{\tiny gt}}]}_{0:2000}$, we make the training dataset ${D}^{[{\mathrm{\tiny train}}]}_{}=\{({\varvec{x}}^{[{\mathrm{\tiny gt}}]}_{t},{y}^{[{\mathrm{\tiny gt}}]}_{t})\mid t \in {I}^{[{\mathrm{\tiny train}}]}_{}\}$ for ${I}^{[{\mathrm{\tiny train}}]}_{}=\{10 \,(=k),11,\ldots ,1999\}$ and ${\varvec{x}}^{[{\mathrm{\tiny gt}}]}_{t}=({y}^{[{\mathrm{\tiny gt}}]}_{t-1},\ldots ,{y}^{[{\mathrm{\tiny gt}}]}_{t-k})^{\mathrm{T}}$. For learning machines $\theta _N$, we have employed single CAN2s ${\theta }^{[{\mathrm {single}}]}_{N}$ and bagging CAN2s ${\theta }^{[{\mathrm{bag}}]}_{N}$ with the number of units $N=5+20i\,(i=0,1,2,\ldots ,14)$. After the training, we execute IOS prediction ${\hat{y}}_{t}={f}^{[\theta _N]}_{}(\varvec{x}_{t})$ for $t=t_p,t_p+1,\ldots$ with the initial input vector $\varvec{x}_{t_{\mathrm{\tiny p}}}=({y}^{[{\mathrm{\tiny gt}}]}_{t_{\mathrm{\tiny p}}-1},\ldots ,{y}^{[{\mathrm{\tiny gt}}]}_{t_{\mathrm{\tiny p}}-k})$ for prediction start time $t_{\mathrm{\tiny p}}\in T_{\mathrm{\tiny p}}=\{2000+100i\mid i=0,1,2,\ldots ,29\}$ and prediction horizon $h_p=500$. We show experimental results for the embedding dimension being $k=10$ and the threshold in (8) being $e_y=10$ (see [7] for the result with $k=8$, which is not significantly but slightly different).

In order to estimate the accuracy of ${y}^{[{\mathrm{\tiny gt}}]}_{t}$, we have obtained an average predictable horizon $\left\langle h\left( {y}^{[{\mathrm{\tiny gt}}]}_{t:500},{y}^{[\Delta t=10^{-5},r=128]}_{t:500}\right) \right\rangle _{t\in T_{\mathrm{p}}}=230$ steps (=5.75 s/25 ms) for the time series ${y}^{[\Delta t=10^{-5},r=128]}_{t:500}$ generated with $\Delta t=10^{-5}$ and $r=128$-bit precision via the Runge–Kutta method. This indicates that ${y}^{[{\mathrm{\tiny gt}}]}_{t}$ with $\Delta t=10^{-4}$ and $r=128$ is considered to be accurate during 230 steps on average because we have observed that predictable horizon of two time series generated by the Runge–Kutta method with step sizes $\Delta t=10^{-n}$ and $10^{-n-1}$ for $n=3,4,5,6,7$ increases monotonically with the decrease in step size or the increase in *n*.

Here, note that we have executed several experiments with using the parameter $\theta =(N,k)$ for $k=6$, 8, 10, 12 and so on, and we do not have found out any critically different results, although we would like to execute and show the results of comparative study in our future research.

### Results and analysis

**Fig. 2:**
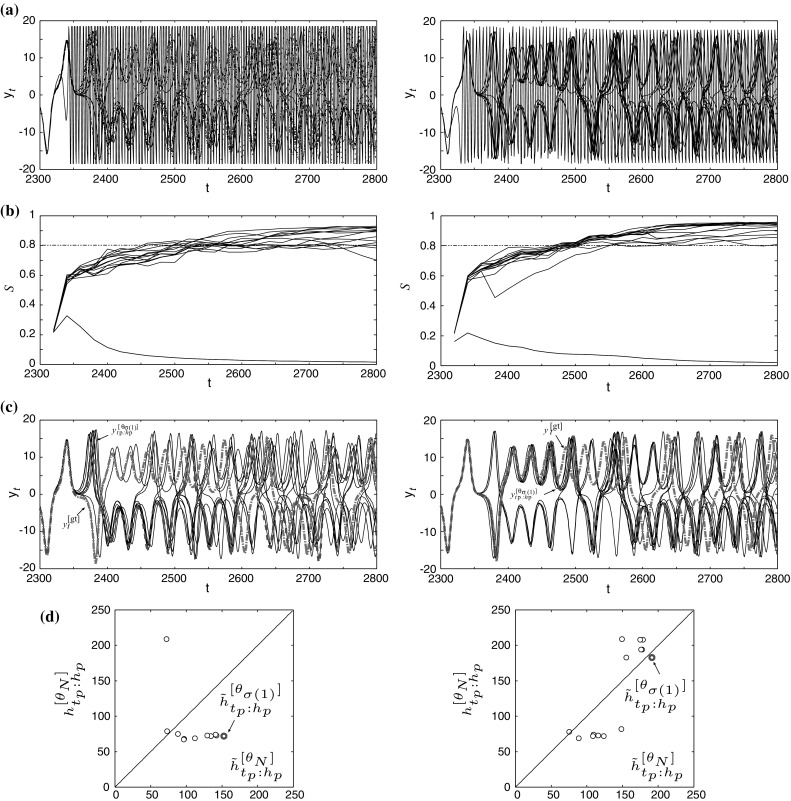
Experimental results obtained by single CAN2s (*left*) and bagging CAN2s (*right*) for the prediction start time $t_p=2300$ and the horizon $h_p=500$. The *top row*, **a**, shows superimposed original predictions ${y}^{[\theta _N]}_{t_p:h_p}$. **b** Shows time evolution of similarity *S* of attractors, and the predictions with $S\ge S_{\mathrm{\tiny th}}=0.8$ at $t=t_p+h_p-1=2799$ are selected as plausible predictions. **c** Shows selected plausible predictions ${y}^{[\theta _N]}_{t_p:h_p}$ as well as ground truth time series ${y}^{[{\mathrm{\tiny gt}}]}_{t}$ (*red*) and representative prediction ${y}^{[\theta _{\sigma (1)}]}_{t_p:h_p}$ (*green*). **d** Shows the relationship between actual predictable horizons ${h}^{[\theta _{N}]}_{t_p:h_p}$ and LOOCV predictable horizons ${\tilde{h}}^{[\theta _{N}]}_{t_p:h_p}$ of plausible predictions (colour figure online)

**Fig. 3:**
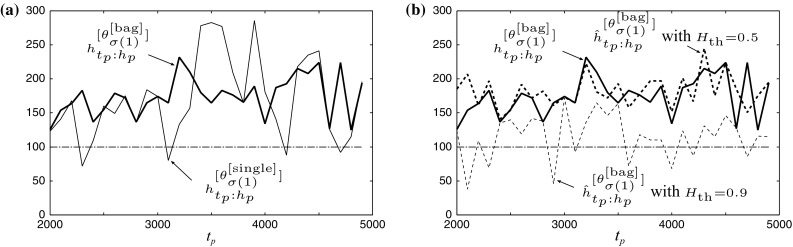
Experimental result of **a** actual predictable horizons ${h}^{[{\theta }^{[{\mathrm {single}}]}_{\sigma (1)}]}_{t_p:h_p}$ and ${h}^{[{\theta }^{[{\mathrm{bag}}]}_{\sigma (1)}]}_{t_p:h_p}$, and **b** estimated predictable horizon ${\hat{h}}^{[{\theta }^{[{\mathrm{bag}}]}_{\sigma (1)}]}_{t_p:h_p}$ with $H_{\mathrm{\tiny th}}=0.9$ and 0.5 for $t_p=2300$. The mean of the predictable horizons is $\left\langle {h}^{[{\theta }^{[{\mathrm {single}}]}_{\sigma (1)}]}_{t_p:h_p}\right\rangle _{t_p\in T_{\mathrm{\tiny p}}}=170$, $\left\langle {h}^{[{\theta }^{[{\mathrm{bag}}]}_{\sigma (1)}]}_{t_p:h_p}\right\rangle _{t_p\in T_{\mathrm{\tiny p}}}=175$, $\left\langle {\hat{h}}^{[{\theta }^{[{\mathrm{bag}}]}_{\sigma (1)}]}_{t_p:h_p}\right\rangle _{t_p\in T_{\mathrm{\tiny p}},H_{\mathrm{\tiny th}}=0.9}=115$ and $\left\langle {\hat{h}}^{[{\theta }^{[{\mathrm{bag}}]}_{\sigma (1)}]}_{t_p:h_p}\right\rangle _{t_p\in T_{\mathrm{\tiny p}},H_{\mathrm{\tiny th}}=0.5}=182$, respectively

**Fig. 4:**
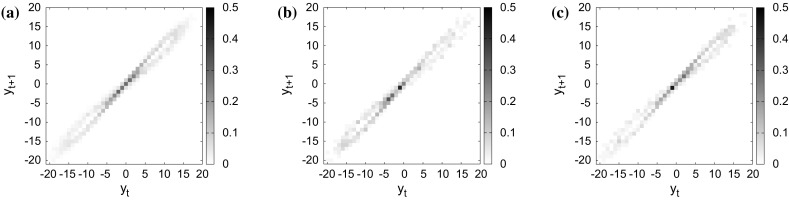
Experimental result of attractor distribution: **a** ${a}^{[{\mathrm{\tiny train}}]}_{ij}$ of training time series ${y}^{[{\mathrm{\tiny train}}]}_{t_g:h_g}$, **b** ${a}^{[{\theta }^{[{\mathrm {single}}]}_{\sigma (1)}]}_{ij}$ of the representative prediction ${y}^{[{\theta }^{[{\mathrm {single}}]}_{\sigma (1)}]}_{t_p:h_p}$ obtained by single CAN2 with $\sigma (1)=N=145$, and **c** ${a}^{[{\theta }^{[{\mathrm{bag}}]}_{\sigma (1)}]}_{ij}$ of the representative prediction ${y}^{[{\theta }^{[{\mathrm{bag}}]}_{\sigma (1)}]}_{t_p:h_p}$ obtained by bagging CAN2 with $\sigma (1)=N=225$, at $t=2799$. The resolution of the distributions is $\Delta _a=(v_{\max }-v_{0})/40=(18.5-(-18.5))/40=0.925$. The similarity $S({y}^{[{\theta }^{[{\mathrm {single}}]}_{\sigma (1)}]}_{t_p:h_p},{y}^{[{\mathrm{\tiny train}}]}_{t_g:h_g})=0.859$ and $S({y}^{[{\theta }^{[{\mathrm{bag}}]}_{\sigma (1)}]}_{t_p:h_p},{y}^{[{\mathrm{\tiny train}}]}_{t_g:h_g})=0.939$

**Fig. 5:**
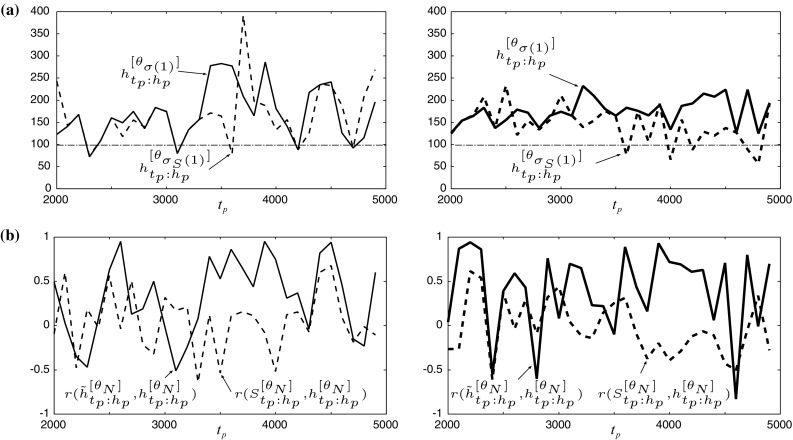
Experimental result of **a** predictable horizons, ${h}^{[\theta _{\sigma (1)}]}_{t_p:h_p}$ and ${h}^{[\theta _{\sigma _S(1)}]}_{t_p:h_p}$, and **b** the correlations $r({\tilde{h}}^{[\theta _N]}_{t_p:h_p},{h}^{[{\theta }_{N}]}_{t_p:h_p})$ and $r({S}^{[\theta _N]}_{t_p:h_p},{h}^{[{\theta }_{N}]}_{t_p:h_p})$ for single CAN2s (*left*) and bagging CAN2s (*right*)

First, we show an example of all predictions ${y}^{[\theta _N]}_{t_p:h_p}$ for $t_p=2300$ in Fig. 2a. Note that $t_p=2300$ is the start time of representative prediction ${y}^{[\theta _{\sigma (1)}]}_{t_p:h_p}$ with predictable horizon ${h}^{[\theta _{\sigma (1)}]}_{t_p:h_p}$ being smaller than 100 by single CAN2 (actually ${h}^{[{\theta }^{[{\mathrm {single}}]}_{\sigma (1)}]}_{t_p:h_p}=72$) and improved by bagging CAN2 as ${{h}}^{[{\theta }^{[{\mathrm{bag}}]}_{\sigma (1)}]}_{t_p:h_p}=183$ (see Fig. 3a).

In Fig. 2b, we can see that single CAN2s have larger number of predictions with the similarity *S* smaller than $S_{\mathrm{\tiny th}}=0.8$ than bagging CAN2s at $t=2799$, and their predictions are not selected as plausible predictions. A detailed analysis of the similarity is shown below.

The representative prediction ${y}^{[\theta _{\sigma (1)}]}_{t_p:h_p}$ (green) shown in (c) is chosen by means of selecting the largest LOOCV predictable horizon ${\tilde{h}}^{[\theta _{\sigma (1)}]}_{t_p:h_p}$ shown in (d). From (d), we can see that the single CAN2 (left) has actual predictable horizon ${h}^{[\theta _N]}_{t_p:h_p}$ larger than 200 and LOOCV predictable horizon ${\tilde{h}}^{[\theta _N]}_{t_p:h_p}$ smaller than 100, actually $({h}^{[\theta _{N}]}_{t_p:h_p},{\tilde{h}}^{[\theta _{N}]}_{t_p:h_p})=(209,72.1)$. Since the present method selects the prediction with the largest ${\tilde{h}}^{[\theta _{N}]}_{t_p:h_p}$, the prediction with ${h}^{[\theta _{N}]}_{t_p:h_p}=209$ could not have selected. On the other hand, we can see that bagging CAN2 (right in (d)) successfully selects the prediction with ${h}^{[\theta _{N}]}_{t_p:h_p}$ larger than 100, actually $({h}^{[\theta _{N}]}_{t_p:h_p},{\tilde{h}}^{[\theta _{N}]}_{t_p:h_p})=(183,191)$. Precisely, bagging CAN2s have successfully provided large ${\tilde{h}}^{[\theta _{N}]}_{t_p:h_p}=191$ because there are a number of predictions with long predictable horizons around ${h}^{[\theta _{N}]}_{t_p:h_p}=200$ as shown as the group of points neighboring ${h}^{[\theta _{N}]}_{t_p:h_p}=200$ in (d) on the right-hand side. Incidentally, from (c), we can see that ensemble mean does not seem appropriate for producing representative prediction in long-term prediction of chaotic time series.

In Fig. 3, we show the results of actual and estimated predictable horizons. Note that we have obtained $\left\langle h\left( {y}^{[{\mathrm{\tiny gt}}]}_{t:500},{y}^{[\Delta t=5\times 10^{-4},r=64]}_{t:500}\right) \right\rangle _{t\in T_p}=172$ steps (=4.3s/25ms) and $\left\langle h\left( {y}^{[{\mathrm{\tiny gt}}]}_{t:500},{y}^{[\Delta t=10^{-3},r=64]}_{t:500}\right) \right\rangle _{t\in T_p}=142$ steps (=3.55 s/25 ms) and the former is almost the same as the mean of predictable horizons achieved by single and bagging CAN2 being 170 and 175 steps, respectively. This indicates that single and bagging CAN2s after learning the training data generated via the Runge–Kutta method with the step size $\Delta t=10^{-4}$ have almost the same prediction performance as the Runge–Kutta method with $\Delta t=5\times 10^{-4}$. Although we do not have no general measure to evaluate time series prediction so far, the above method using the step size of Runge–Kutta method and the mean predictable horizon seems reasonable. In Fig. 3a, we can see that the performance of the stability of prediction by single CAN2 is improved by bagging CAN2 from the point of view that the former has four actual predictable horizons ${h}^{[{\theta }^{[{\mathrm {single}}]}_{\sigma (1)}]}_{t_p:h_p}$ smaller than 100 among all predictions for $t_{\mathrm{\tiny p}}\in T_{\mathrm{\tiny p}}$ and bagging CAN2 has achieved all ${h}^{[{\theta }^{[{\mathrm{bag}}]}_{\sigma (1)}]}_{t_p:h_p}$ larger than 100. From (b), we can see that the estimated predictable horizon ${\hat{h}}^{[\theta _{\sigma (1)}]}_{t_p:h_p}$ with $H_{\mathrm{\tiny th}}=0.5$ is almost the same as actual predictable horizon ${h}^{[\theta _{\sigma (1)}]}_{t_p:h_p}$, while $H_{\mathrm{\tiny th}}=0.9$ has achieved safe estimation, or ${\hat{h}}^{[\theta _{\sigma (1)}]}_{t_p:h_p}\le {h}^{[\theta _{\sigma (1)}]}_{t_p:h_p}$,

In order to analyze the property of the method, we show the attractor distribution of training and representative time series in Fig. 4. We can see that the similarity of attractors $S({y}^{[{\theta }^{[{\mathrm {single}}]}_{\sigma (1)}]}_{t_p:h_p},{y}^{[{\mathrm{\tiny train}}]}_{t_g:h_g})=0.859$ obtained by single CAN2 is smaller than $S({y}^{[{\theta }^{[{\mathrm{bag}}]}_{\sigma (1)}]}_{t_p:h_p},{y}^{[{\mathrm{\tiny train}}]}_{t_g:h_g})=0.939$ obtained by bagging CAN2. From the result on the left in Fig. 2b, we can see that there is a prediction with the similarity larger than 0.859 for single CAN2. Actually, the maximum similarity of single CAN2s is 0.931. The prediction ${y}^{[{\theta }_{\sigma _S(1)}]}_{t_p:h_p}$ with the maximum similarity of attractors in plausible predictions has a possibility to be used for selecting a representative prediction, where $\theta _{\sigma _S(1)}$ indicates the learning machine with the maximum similarity. The comparison between ${h}^{[\theta _{\sigma (1)}]}_{t_p:h_p}$ and ${h}^{[\theta _{\sigma _S(1)}]}_{t_p:h_p}$ is shown in Fig. 5a, where ${h}^{[\theta _{\sigma _S(1)}]}_{t_p:h_p}$ seems competitive with ${h}^{[\theta _{\sigma (1)}]}_{t_p:h_p}$ for single CAN2, but worse for bagging CAN2. To analyze much more, we have examined the correlation $r({S}^{[\theta _N]}_{t_p:h_p},{h}^{[{\theta }_{N}]}_{t_p:h_p})$ between the similarity ${S}^{[\theta _N]}_{t_p:h_p}=S({y}^{[{\theta }_{N}]}_{t_p:h_p},{y}^{[{\mathrm{\tiny train}}]}_{t_g:h_g})$ and the predictable horizon ${{h}}^{[\theta _{N}]}_{t_p:h_p}=h({y}^{[{\theta }_{N}]}_{t_p:h_p},{y}^{[{\mathrm{\tiny train}}]}_{t_g:h_g})$, as well as the correlation $r({\tilde{h}}^{[\theta _N]}_{t_p:h_p},{h}^{[{\theta }_{\sigma _S(1)}]}_{t_p:h_p})$ as shown in Fig. 5b. From this result, there are a number of cases with positive low or negative value of correlations. In particular, the correlation of similarity, $r({S}^{[\theta _N]}_{t_p:h_p},{h}^{[{\theta }_{N}]}_{t_p:h_p})$, has few cases with the values larger than 0.5 for both single and bagging CAN2. This suggests that the selection of representative prediction by using the similarity measure is not so reliable. On the other hand, bagging CAN2 has larger number of cases with the correlations larger than 0.5 as we can see the thick line of $r({\tilde{h}}^{[\theta _N]}_{t_p:h_p},{h}^{[{\theta }_{\sigma _S(1)}]}_{t_p:h_p})$ on the right-hand side in Fig. 5b. Furthermore, we can see that there are several cases of $t_p$ with negative correlations $r({\tilde{h}}^{[\theta _N]}_{t_p:h_p},{h}^{[{\theta }_{\sigma _S(1)}]}_{t_p:h_p})$ in (b), and the corresponding predictable horizons ${h}^{[\theta _{\sigma (1)}]}_{t_p:h_p}$ in (a) are shorter than the neighboring (w.r.t. $t_p$) horizons. This correspondence seems reasonable because negative correlation does not contribute to the selection of the prediction with large predictable horizon. Thus, we have to remove the cases of negative correlations. So far, we have two approaches: one is to improve the performance of learning machine much more as we have done with the bagging method in this paper, and the other is to refine the selection method by means of modifying LOOCV predictable horizon or developing new methods. Actually, we have predictions with much longer predictable horizons not shown in this paper, but we cannot select such predictions without knowing the ground truth time series, so far.

## Conclusion

We have presented a performance improvement in the method for probabilistic prediction of chaotic time series by means of using bagging learning machines. The method obtains a set of plausible predictions by means of using similarity of attractors between training and predicted time series. And then, it provides representative prediction which has the longest LOOCV predictable horizon. By means of executing numerical experiments using single and bagging CAN2s, we have shown that bagging CAN2 improves the performance of single CAN2 and analyzed the relationship between LOOCV and actual predictable horizons. In our future research studies, we would like to overcome the problem of negative correlation between the achieved predictable horizon and the LOOCV predictable horizon, or the measure of selecting representative prediction.
